# Lactoyl leucine and isoleucine are bioavailable alternatives for canonical amino acids in cell culture media

**DOI:** 10.1002/bit.27755

**Published:** 2021-04-08

**Authors:** Corinna Schmidt, Maria Wehsling, Maxime Le Mignon, Gregor Wille, Yannick Rey, Alisa Schnellbaecher, Dmitry Zabezhinsky, Markus Fischer, Aline Zimmer

**Affiliations:** ^1^ Merck Life Science, Upstream R&D Darmstadt Germany; ^2^ Merck Life Science, Process Development Buchs Switzerland

**Keywords:** bioprocesses, cell culture media, enzymatic cleavage, lactoyl‐ (iso)leucine, solubility

## Abstract

Increasing demands for protein‐based therapeutics such as monoclonal antibodies, fusion proteins, bispecific molecules, and antibody fragments require researchers to constantly find innovative solutions. To increase yields and decrease costs of next generation bioprocesses, highly concentrated cell culture media formulations are developed but often limited by the low solubility of amino acids such as tyrosine, cystine, leucine, and isoleucine, in particular at physiological pH. This study sought to investigate highly soluble and bioavailable derivatives of leucine and isoleucine that are applicable for fed‐batch processes. *N*‐lactoyl‐leucine and *N*‐lactoyl‐isoleucine sodium salts were tested in cell culture media and proved to be beneficial to increase the overall solubility of cell culture media formulations. These modified amino acids proved to be bioavailable for various Chinese hamster ovary (CHO) cells and were suitable for replacement of canonical amino acids in cell culture feeds. The quality of the final recombinant protein was studied in bioprocesses using the derivatives, and the mechanism of cleavage was investigated in CHO cells. Altogether, both *N*‐lactoyl amino acids represent an advantageous alternative to canonical amino acids to develop highly concentrated cell culture media formulations to support next generation bioprocesses.

## Graphical abstract

The replacement of leucine and isoleucine by *N*‐lactoyl amino acids in concentrated feeds enables the development of next generation fed‐batch bioprocesses used to produce monoclonal antibodies or recombinant proteins.

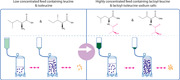

## 1 INTRODUCTION

Therapeutic proteins against cancer, autoimmune, and infectious diseases are being produced in mammalian cell culture (Aggarwal, 2014; Grilo & Mantalaris, 2019) and continuous competition in the biopharmaceutical field leads to increasing demand for biotherapeutics (O'Flaherty et al., 2020). The majority of all biotherapeutics (60%–70%) are produced in cultured Chinese hamster ovary (CHO) cells, due to their capacity to process complex glycosylation, and other human‐like posttranslational modifications (Grilo & Mantalaris, 2019; McAtee et al., 2014; Wurm, 2004). In addition, CHO cells can be grown in suspension in chemically defined media at scales required to meet market demands (Lai et al., 2013).

The cell culture medium (CCM) formulation is critical for the culture longevity and viability as well as quality attributes of the end product (D. Bruhlmann et al., 2015; David Brühlmann et al., 2017; Ehret et al., 2019; Zimmermann et al., 2019). To grow and produce high amounts of antibodies or other therapeutic proteins, CHO cells need regular supplementation with essential amino acids (AA) through feeding (Carrillo‐Cocom et al., 2015; Sellick et al., 2011). However, several amino acids such as cystine, tyrosine and branched‐chain amino acids (BCAA) present an intrinsic low solubility in CCM (Carta & Tola, 1996; Salazar et al., 2016). Several studies report utilization of noncanonical amino acids for biotechnological applications to support growth and productivity of CHO cells while presenting increased solubility properties in CCM (Chevallier et al., 2021; Hecklau et al., 2016; Schmidt et al., 2020; Wu et al., 2008; Zimmer et al., 2014).

The BCAA leucine (Leu), isoleucine (Ile), and valine (Val) are essential amino acids, that cells cannot produce from building blocks (Monirujjaman & Ferdouse, 2014; Tajiri & Shimizu, 2018). These amino acids have interconnected effects and implications on health (Son et al., 2020; Suryawan et al., 2008; Yoneshiro et al., 2019), diseases (Lei et al., 2020), and antibody productivity (Carrillo‐Cocom et al., 2015; Sellick et al., 2011).

*N*‐Lactoyl amino acids (Lac‐AA) were discovered recently as being formed during fermentation of Parmigiano‐Reggiano cheese (Sforza et al., 2009) and in dry‐cured ham (Paolella et al., 2018). Further studies in the food industry described Lac‐AA as taste active amino acids, formed from free AA and lactic acid through the action of lactoyl transferase in *lactobacillus spp*., particularly in soy sauce and meat products (Frerot & Chen, 2013; Zhao et al., 2016). In fermented soy sauce, the most prevalent Lac‐AA were *N*‐lactoyl‐glutamate, *N*‐lactoyl‐valine, *N*‐lactoyl‐isoleucine (Lac‐Ile), and *N*‐lactoyl‐leucine (Lac‐Leu) (Frerot & Chen, 2013). In another study, *N*‐lactoyl‐glutamate, *N*‐lactoyl‐alanine, and *N*‐lactoyl‐methionine were described as ingredients for the preparation of flavored food products upon which they confer a remarkable fullness and mouthfeel (Frerot & Escher, 1998). Lac‐AA and in particular *N*‐Lactoyl‐serine have furthermore been patented for their use in cosmetic and dermatology applications (Haider, 1996). In the animal nutrition field, Lac‐AA were used as supplement to provide ruminants with bioavailable AA that are resistant to degradation in the rumen (Lorbert et al., 2004). Finally, a recent study demonstrated the ability of mammalian cells to produce detectable amounts of lactoyl amino acids intracellularly via cytosolic nonspecific dipeptidase 2 (CNDP2), and subsequent excretion via the ATP‐binding cassette subfamily C member 5 (ABCC5) transporter (Jansen et al., 2015).

This study reports that Ile and Leu can be replaced in feed formulations by their modified analogs Lac‐Leu and Lac‐Ile sodium salts. These modified AA are soluble, stable, and bioavailable for cells when cultivated in fed‐batch mode. Mechanistic studies showed that Lac‐AA are cleaved by the intracellular enzymatic machinery, forming canonical and readily bioavailable Leu or Ile which are being used as building blocks for antibody and fusion protein production or used as precursors for metabolic intermediates.

## 2 MATERIAL AND METHODS

### 2.1 Reagents/cell lines

*N*‐lactoyl‐leucine (Frerot & Escher, 1998) and *N*‐lactoyl‐isoleucine sodium salts depicted in Figure S1 were synthesized according to published protocols (Jörres et al., 1998). 1H (400.2 MHz) and 13C (100.6 MHz) NMR spectra were recorded on a Bruker Avance III 400 spectrometer in D_2_O with benzene‐d_6_ as external standard unless otherwise indicated and are reported in ppm (Figure S1). Mass spectroscopy was performed on an Impact II (Bruker) using electrospray ionization (ESI), details of the method can be found in Section 2.3.

All other chemicals were purchased from Sigma‐Aldrich/Merck (Merck) unless stated otherwise. Five CHO cell lines producing three different IgGs and two fusion proteins were used in this study. Cell line 1 is a recombinant CHOK1 GS clone expressing mAb1, whereas Cell line 2 is a CHODG44 clone expressing mAb2. Cell lines 3, 4, and 5 are CHOZN clones expressing mAb3 and two different fusion proteins and are named CHOZN 023, CHOZN 034, and CHOZN 5, respectively.

### 2.2 Solubility of Lac‐AA

The maximum solubility of Lac‐Ile and Lac‐Leu in water was assessed at different temperatures through the preparation of a saturated solution. The pH value of this solution was recorded. After sedimentation, the supernatant was transferred to a new vial, the aqueous solvent was evaporated using infrared (120°C, 120 min), the residual mass was determined, and final solubility was calculated in g compound per kg of water.

To investigate the solubility of Lac‐Ile and Lac‐Leu in a highly concentrated cell culture feed, a customized dry powder media corresponding to Cellvento® 4Feed lacking Ile and Leu was produced (Merck). Increasing amounts of Lac‐Ile and Lac‐Leu were added to the liquid feed containing already a total of 125 g/L nutrients at pH 7.0 until saturation was observed. After each addition, the feed was agitated for 10 min, and osmolality (Osmomat 3000, GonoTec), pH and turbidity (Turb® 550 IR, WTW Lab, Xylem Analytics) were measured.

To determine the maximum solubility that can be achieved for a feed upon the replacement of Leu and Ile with Lac‐Leu and Lac‐Ile respectively, a Cellvento® 4Feed backbone lacking Leu and Ile was supplemented with Lac‐Leu and Lac‐Ile and compared with the original Cellvento® 4Feed formulation containing Leu and Ile. Both formulations were reconstituted at 230 g/L and 265 g/L, respectively. After 15 min, the pH was adjusted to 7.0 using NaOH and the solution was mixed another 30 min. Increasing amounts of water were added until a clear solution was obtained with a turbidity value below 5 NTU (measured using Turb® 550 IR). Samples were taken before and after each of the dilution steps to measure turbidity.

### 2.3 Stability of Lac‐AA

To monitor the stability of Lac‐AA in the complex feed mixture, a targeted liquid chromatography‐mass spectrometry (LC–MS) method was developed. Lac‐Leu and Lac‐Ile concentrations from 100 μM to 100 mM in Cellvento® 4Feed lacking Ile and Leu at pH 7 were measured to determine the linear range of the method. The final linear range was defined between 10 and 400 pmol of both Lac‐AA injected on the column. Samples were injected on a Xselect HSS T3 column (Waters, 3.5 μm, 2.1 × 150 mm) heated at 40°C. The elution was performed using a gradient between buffer A (20 mM ammonium formate/0.1% formic acid) and buffer B (methanol) using a flow rate of 300 μl/min. Briefly, the gradient started with 0.1% of B for 2 min, then increased linearly from 0.1% B to 20% B at 4 min, followed by 30% B at 6 min, 80% B at 8 min, and 100% B at 8.5 min. A plateau at 100% B was maintained until 9.5 min before returning to 0.1% B until 12 min. The MS detection was performed using an Impact II (Bruker) running in ESI negative mode with an end plate offset of 500 V and a capillary voltage of 3500 V. The nebulizer was set to 1.4 bar and the dry gas was set to 9 L/min at a temperature of 250°C. The mass range was set from 20 to 1000 Da at a spectral rate of 5 Hz. Internal calibration was performed using a sodium formate solution. For the stability study, the cell culture feed (Cellvento® 4Feed lacking Ile/Leu) supplemented with Lac‐AA was reconstituted, the pH was adjusted to 7.0 and the solution was stored for 3 months at either 4°C or room temperature (RT), light protected. Every week, samples were collected and stored at −20°C for further analytics including LC–MS. The signal for Lac‐Leu and Lac‐Ile was considered stable if the measured concentrations over time did not decrease beyond 10% (standard error of the method).

### 2.4 Fed‐Batch and corresponding analytics

CHOK1 GS and CHODG44 cells were cultivated in the Cellvento® 4CHO medium, whereas CHOZN 023, CHOZN 5, and CHOZN 034 were cultivated in EX‐CELL® Advanced™ CHO Fed‐batch Medium (Merck/Sigma Aldrich). Both media are chemically defined and contain essential components for optimal cell growth and protein production. CHOK1 GS and CHOZN cells were transfected with a plasmid containing the Glutamine Synthetase (GS) gene and thus did not require l‐glutamine supplementation. In contrast, CHODG44 cells required supplementation with 6 mM l‐glutamine in the medium. Regarding feeds, Cellvento® 4Feed and EX‐CELL® Advanced feed were lacking Ile and Leu to allow individual supplementation or exchanges with Lac‐Leu and Lac‐Ile (equimolar concentrations or decreasing concentrations going from 100% to 50% the equimolar concentration). Fed‐batch processes were performed in 50 ml spin tubes with vented cap (TPP) at 37°C, 5% CO_2_, 80% humidity and a rotation speed of 320 rpm. For Cellvento® 4Feed, 3% (v/v) of the total volume was added on Day 3, 5, 10, 12, and 14 and 6% (v/v) was added on Day 7. For EX‐CELL® Advanced based processes, 5% (v/v) of EX‐CELL® Advanced™ Feed was added on Day 3, 5, 7, 10, 12, and 14. The glucose level was maintained by adding a specific amount of a 400 g/L glucose stock solution on demand to up to 6 g/L during the week and up to 12 g/L before the weekend. Cell counts and viability were measured using a Vi‐CELL™XR 2.04 cell counter (Beckman Coulter). The seeding density was 2 × 10^5^ cells/ml for CHOK1 GS and CHODG44 and 3 × 10^5^ cells/ml for CHOZN cells in 30 ml starting volume. Experimental conditions were performed with four biological replicates. Spent media analysis including glucose, IgG, lactate dehydrogenase, ammonia, iron, lactate, phosphate, and pyruvate was performed with the bioprocess analyzer Cedex Bio HT (Roche) after centrifugation of the sample for 2.5 min at 5000 rpm (2541 g).

For bioreactor experiments using the CHOK1 GS clone, DasGIP 1.2 L glass bioreactors were used, pH was controlled at 6.95 ± 0.15, and dissolved oxygen (DO) was controlled at 50% air saturation by sparging with air and pure oxygen gas via an open pipe sparger. Temperature and agitation were set to 36.7 C and 140 rpm, respectively. The control condition was fed with classical Cellvento® 4Feed (containing Ile and Leu) at 1x (same feeding regime as above). The Lac‐AA containing feed was designed based on the small‐scale dose finding experiment (70% Lac‐Leu and Lac‐Ile) and was concentrated 2x allowing to reduce the volume of feed added to the bioreactor by a factor 2. For the concentrated process, 1.5% (v/v) of feed was added on Day 3, 5, 10, 12, and 14 and 3% (v/v) on Day 7. To see the potential benefit of a process using highly concentrated feeds, the starting volume of the Lac‐AA bioreactors was adapted to maximize the bioreactor capacity and the process started with a 12% higher volume compared to the control condition (same seeding density).

Amino acid analysis was performed by Ultra‐Performance Liquid Chromatography (UPLC) with an AccQ‐Tag Ultra C18 column (Waters). To detect AA using an UV detector, samples were derivatized with the AccQ‐Tag Ultra Derivatization Kit (Waters). Quantification was performed using external calibration curves according to the manufacturer protocol. Lac‐AA were quantified using the LC‐MS method described in paragraph 2.3 and using an isotopically labeled standard (synthesized internally).

### 2.5 Antibody purification and cQA analysis

Antibodies and fusion proteins were purified from the cell culture supernatant using protein A PhyTips® (PhyNexus Inc.). Glycosylation patterns were analyzed either by capillary gel electrophoresis with laser‐induced fluorescence (CGE‐LIF) or by UPLC coupled to a mass spectrometer as described elsewhere (Ehret et al., 2019). Distribution of charge variants was determined by capillary isoelectric focusing using the CESI8000 (Sciex) and the corresponding manufacturer protocol. Aggregates were quantified using size exclusion chromatography coupled to a Diode array detector (214 nm) using an Acquity UPLC (Waters) and a TSK gel Super SW series column at RT (Tosoh Bioscience). The mobile phase was a 0.05 M sodium phosphate, 0.4 M sodium perchlorate solution at pH 6.3 and the flow rate was 0.35 ml/min. The sample concentration was adjusted to 1 mg/ml with storage buffer (85% v/v of 30 mM citric acid pH 3.0 and 15% v/v 0.375 M Tris Base pH 9.0) and 10 µl were injected for quantification.

### 2.6 Cleavage of Lac‐AA

CHOK1 GS cell lysates were obtained by incubation of cell pellets in CytoBuster™ Protein Extraction Reagent (Cat#71009‐M, Merck) as indicated in the manufacturer instructions and stored at −20°C. Protein concentrations were determined using Pierce™ BCA Protein Assay Kit (Cat# 23227, Thermo Fisher Scientific). Lysates were diluted with 10 mM Tris HCl (pH 7.4) to obtain protein concentrations of 37.5, 75, 150, and 300 μg/ml and mixed with 25 mM Lac‐Leu or Lac‐Ile in 10 mM Tris⋅HCl and 50 mM NaCl (pH 7.4) buffer. Samples were collected at different time points after incubation at 20°C or 37°C. The reaction was stopped by incubation at 100°C for 10 min and kept at −20°C before performing the lactate measurement using the lactate fluorescent assay kit (Cat# MAK064, Merck) or amino acid analysis using the AccQ‐Tag method described above.

Recombinant human CNDP2 (1 μg, C‐terminal HIS‐tagged; Prospec) was incubated (37°C) with 10 mM Lac‐Leu or Lac‐Ile in 25 mM Tris⋅HCl (pH 7.4), containing 0.1 mM MnCl_2_. The reactions were stopped by incubation at 100°C for 10 min and kept at −20°C before performing lactate and AA measurements.

### 2.7 Proteomic analysis of CHOK1 GS cells

#### 2.7.1 Preparation of cell lysates

Cell pellets from a fed‐batch experiment using either the control feed, or a feed containing Lac‐Leu and Lac‐Ile, were collected at Day 10 by centrifugation at 270 g for 5 min. The cell pellet was resuspended in Cytobuster (Merck) at a concentration of 1 × 10^7^ cells per 100 µl and incubated for 10 min at RT. The lysis mix was centrifuged at 15,000 g (4°C) for 5 min, and the supernatants were transferred in 100 µl aliquots to 1.5 ml Eppendorf tubes and stored at −20°C.

#### 2.7.2 Protein precipitation

Proteins were precipitated using a TCA/acetone protocol. Briefly, 100 µl lysate were mixed with 20% TCA/80% acetone/10 mM DTT in a 1:1 ratio (v:v) and incubated at −20°C for 2 h. Then, pellets were resuspended with 40–80 µl of 8 M urea, 25 mM Tris‐HCl, pH 8 and then buffer exchanged in 100 mM Tris‐HCl, pH 8 using Zeba spin desalting column (Thermo Fisher Scientific). Protein quantification was performed using NanoDrop (Thermo Fisher Scientific).

#### 2.7.3 Tryptic digestion protocol

Samples were digested using trypsin before nanoLC‐MS/MS analysis. Briefly, 30 µg of intracellular proteins were denatured and reduced by adding 1 µl of both 1% ProteaseMAX (Promega) and 250 mM DTT, respectively, followed by an incubation at 56°C for 60 min in a thermomixer (Thermo Fisher Scientific, 600 rpm). After cooling down the samples at RT, free cysteines were alkylated by adding 2 µl of 250 mM iodoacetamide. After an incubation at RT for 45 min in the dark, excess of iodoacetamide was quenched by adding 1 µl of 250 mM DTT. 61 µl of 100 mM Tris HCl, pH 8, 1 µl of 1 M CaCl_2_, and 2 µl of trypsin Gold at 0.5 µg/µl (Promega) were successively added. After an incubation at 37°C for 15 h, enzymatic digestion was stopped by adding 1 µl of 100% formic acid. A centrifugation step (14,000 rpm for 15 min) was performed to remove degraded surfactant and 50 µl of supernatant were transferred in a LC vial containing 50 µl of 4% ACN/0.2% TFA.

#### 2.7.4 LC‐MS parameters

1 µg of tryptic digest was injected on a nanoRSLC3000 (Thermo Fisher Scientific) coupled to QToF Impact II equipped with a Captive spray (Bruker). For the LC, an Acclaim PepMap trap column (100 µm × 2 cm) was used together with a nanoEase M/Z Peptide CSH C18 column (130 Å, 1.7 µm, 75 µm × 150 mm). The column was heated at 40°C, two solvents were used (A: 0.1% formic acid, B: 80% ACN/0.1% formic acid) at a flow rate of 300 nl/min. The gradient was raised stepwise from 5% B to 95% B according to the following gradient (0 min 95:5, 5 min 95:5, 102 min 60:40, 105 min 40:60, 106 min 5:95, 110 min 5:95, 110.1 min 95:5, and 120 min 95:5). The mass spectrometer was run in positive mode for a m/z range from 150 to 2200 with lock mass calibration (m/z 1221.9906). The capillary voltage was set to 1100 V, the MS spectra rate was 2 Hz whereas the MSMS scan level was 8 Hz (2500 cts); 32 Hz (25000 cts).

#### 2.7.5 Peptide/protein identification

Protein identification was performed using PEAKS Xpro software (BSI Informatics) by matching acquired peptide tandem mass spectra against the *Cricetulus griseus* UniprotKB proteome database (downloaded on 29/10/2020, 56,563 entries). First, data (.mgf) were converted by Progenesis QI for Proteomics software (Waters) before loading into PEAKS Xpro software. Database search was performed using the following parameters: peptide tolerance of 10 ppm, fragment ion tolerance of 0.05 Da, trypsin enzyme (specific), two missed cleavages allowed, carbamidomethylation of cysteine as a fixed modification, methionine oxidation, and asparagine/glutamine deamidation as variable modifications, allowing a maximum of two variable modifications per peptide. Only proteins identified with at least one unique peptide (FDR < 1% at the peptide level) were validated.

#### 2.7.6 Label free MS quantification

Label‐free MS quantification was performed using Progenesis QI for Proteomics software. Raw data were loaded as centroided data (resolution 40,000) without additional calibration (previously performed by Data Analysis software, Bruker). “All proteins” method was used for normalization and only proteins quantified with at least three unique peptides were considered. Only proteins with a fold change > 2 and a *q*‐value < 0.01 were considered as statistically significant. All proteins were plotted in a volcano plot according to their Log2(fold change) and −Log10(*q*‐value) using GraphPad prism 7.0 (GraphPad Software Inc.).

## 3 RESULTS

### 3.1 Solubility of Lac‐AA

To determine whether Lac‐AA present a benefit compared to canonical AA, the solubility of Leu, Ile, and their respective *N*‐lactoyl‐amino acids, Lac‐Leu, and Lac‐Ile, was determined in water using saturated solutions and residual mass determination at 4, 20, 25, and 37°C. As shown in Table S1, the solubility of Lac‐Leu and Lac‐Ile was significantly higher when compared with the solubility of the respective canonical AA in water. For instance, the maximum solubility of Leu and Leu sodium salt at 25°C was 22.1 g/kg (pH 6.0) and 86.0 g/kg (pH 10.8), respectively, whereas the solubility of Lac‐Leu sodium salt was 689.2 g/kg (pH 6.7). For Ile and Lac‐Ile sodium salt, solubilities of 32.4 and 639.3 g/kg were observed at 25°C with a pH of 6.2 and 6.14, respectively. Altogether, these results indicate that Lac‐Leu and Lac‐Ile salts are suitable candidates to increase the solubility of cell culture media and feed formulations by replacement of their respective canonical AA. To confirm that Lac‐Leu and Lac‐Ile salts are highly soluble in complex CCM formulations at neutral pH, increasing amounts of Lac‐AA were added to Cellvento® 4Feed lacking Ile and Leu. Similarly, increasing amounts of Ile and Leu were added to the same feed formulation as a control (Figure S2). The pH was always adjusted to 7.0 ± 0.2 before each measurement. The maximum solubility of Leu and Ile in Cellvento® 4Feed lacking Ile/Leu was found to be 90 mM and 105 mM, respectively. In contrast, the maximum soluble concentration (with a turbidity value below 5 NTU) of Lac‐Leu was 590 mM, indicating that the Lac‐AA is at least five times more soluble than Leu in the feed. For Lac‐Ile, the maximum tested concentration of 950 mM was still soluble in the feed with a turbidity value below 5 NTU. This indicates that Lac‐Ile is at least nine times more soluble than Ile in neutral pH Cellvento® 4Feed lacking Ile/Leu.

Further experiments were designed to determine the maximum concentration that can be reached for a complex CCM formulation when Leu and Ile are replaced by their respective lactoyl derivatives. Data for Cellvento® 4Feed (containing Ile and Leu) indicate that this formulation is insoluble at already 1.2× (160 g/L—turbidity value around 50 NTU). In contrast, if Leu and Ile are replaced with their equimolar concentration of Lac‐Leu and Lac‐Ile, the formulation is soluble until 200 g/L which indicates that the replacement of Leu and Ile by their respective *N*‐lactoyl counterparts allows to increase by 54% the total concentration of a complex CCM formulation.

### 3.2 Stability of Lac‐AA

Limited reports are available about the stability of Lac‐AA. Therefore, a new targeted LC‐MS method was developed to monitor the stability of Lac‐AA in complex feed mixtures. The method proved to be linear between 10 pmol and 400 pmol of Lac‐AA injected on the column and was used to monitor the stability of Lac‐Leu and Lac‐Ile in Cellvento® 4Feed lacking Ile and Leu and stored for 3 months, light protected, at either 4°C or RT. Results (Figure S3) indicate that the concentration of both Lac‐AA stayed within the limit of ± 10% of the starting concentration during the whole storage period which indicates an overall good stability at both storage temperatures.

### 3.3 Bioavailability of Lac‐AA in fed‐batch processes

To understand whether Lac‐AA derivatives can replace Ile and Leu in feeds, small scale fed‐batch experiments were performed in spin tubes with five distinct CHO cell clones. The positive control feed contained Ile and Leu and was compared to three different test feeds: the first feed contained Lac‐Leu and Ile, the second contained Lac‐Ile and Leu, and the third feed contained Lac‐Leu and Lac‐Ile. Equimolar ratios of Lac‐Leu and Lac‐Ile were added to the feed to match the concentrations of the canonical AA. The negative control feed was lacking Leu and Ile and was used to monitor the overall Leu and Ile consumption from the basal growth medium.

**Figure 1:**
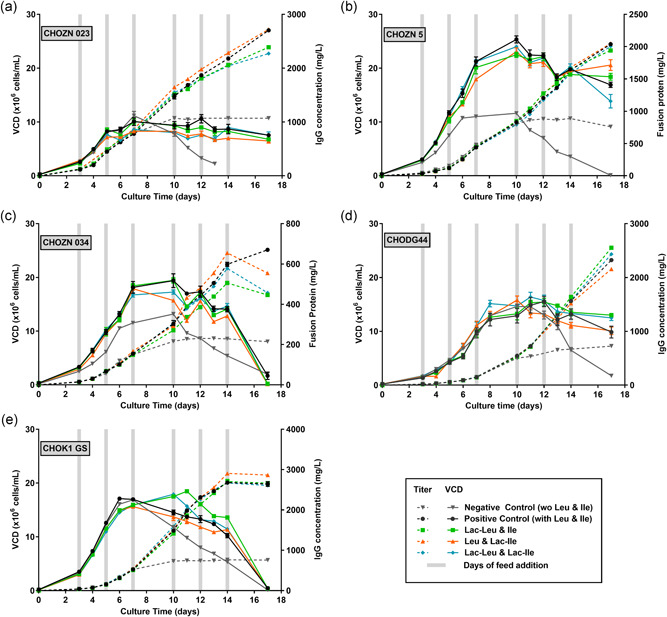
Viable cell density and titer obtained in fed‐batch with control feeds or feeds containing Lac‐Leu, Lac‐Ile, or Lac‐Leu and Lac‐Ile for five cell lines. The positive control feed contained Leu and Ile, the negative control feed was lacking Leu and Ile, the test feeds were prepared by replacing Leu and Ile individually or in combination by their equimolar concentration of Lac‐Ile and Lac‐Leu. (a)–(c) CHOZN 023, CHOZN 5, and CHOZN 034 cells were cultivated in EX‐CELL® Advanced medium and 5% (v/v) of modified EX‐CELL® Advanced™ Feed was added on Day 3, 5, 7, 10, 12, and 14. (d) CHODG44 cells were cultivated in Cellvento® 4CHO supplemented with 6 mM l‐glutamine and 1.5% (v/v) of modified Cellvento® 4Feed was added on Day 3 and 3% on Days 5, 7, 10, 12, and 14. (e) CHOK1 GS were cultivated in Cellvento® 4CHO medium and 3% (v/v) of modified Cellvento® 4Feed was added on Day 3, 5, 10, 12, and 14 and 6% (v/v) on Day 7. Cultivation was performed in 50 mL spin tubes at 37°C, 5% CO_2_, 80% humidity, and a rotation speed of 320 rpm. VCD (plain lines) were measured using Vi‐CELL™XR and titer (dotted lines) was measured using Cedex Bio HT. Data represent mean ± *SEM* of a minimum of four biological replicates. CHO, Chinese hamster ovary; Ile, isoleucine; Leu, leucine; *SEM*, standard error of mean [Color figure can be viewed at wileyonlinelibrary.com]

Results shown in Figure 1 indicate that independently of the cell lineage (CHOZN in Figure 1a–c; CHODG44 in Figure 1d and CHOK1 GS in Figure 1e) and the type of recombinant protein produced (three distinct IgG1 mAbs in Figures 1a, 1d, and 1e or two distinct fusion proteins in Figures 1b and 1c), neither growth nor titer were impacted by the replacement of Leu and Ile by their Lac‐AA counterparts in the feed. In contrast, viable cell density (VCD) decreased rapidly after Day 7 or Day 10 when cells were fed with feed lacking Leu and Ile, indicating that these AA are essential for growth and survival as already known from previous reports (Zhang et al., 2017). Importantly, in this condition, the production of the recombinant protein of interest was drastically reduced after Day 7 or Day 10 for all the five tested cell lines, confirming the requirement for an external supply of BCAA through feeds.

**Figure 2:**
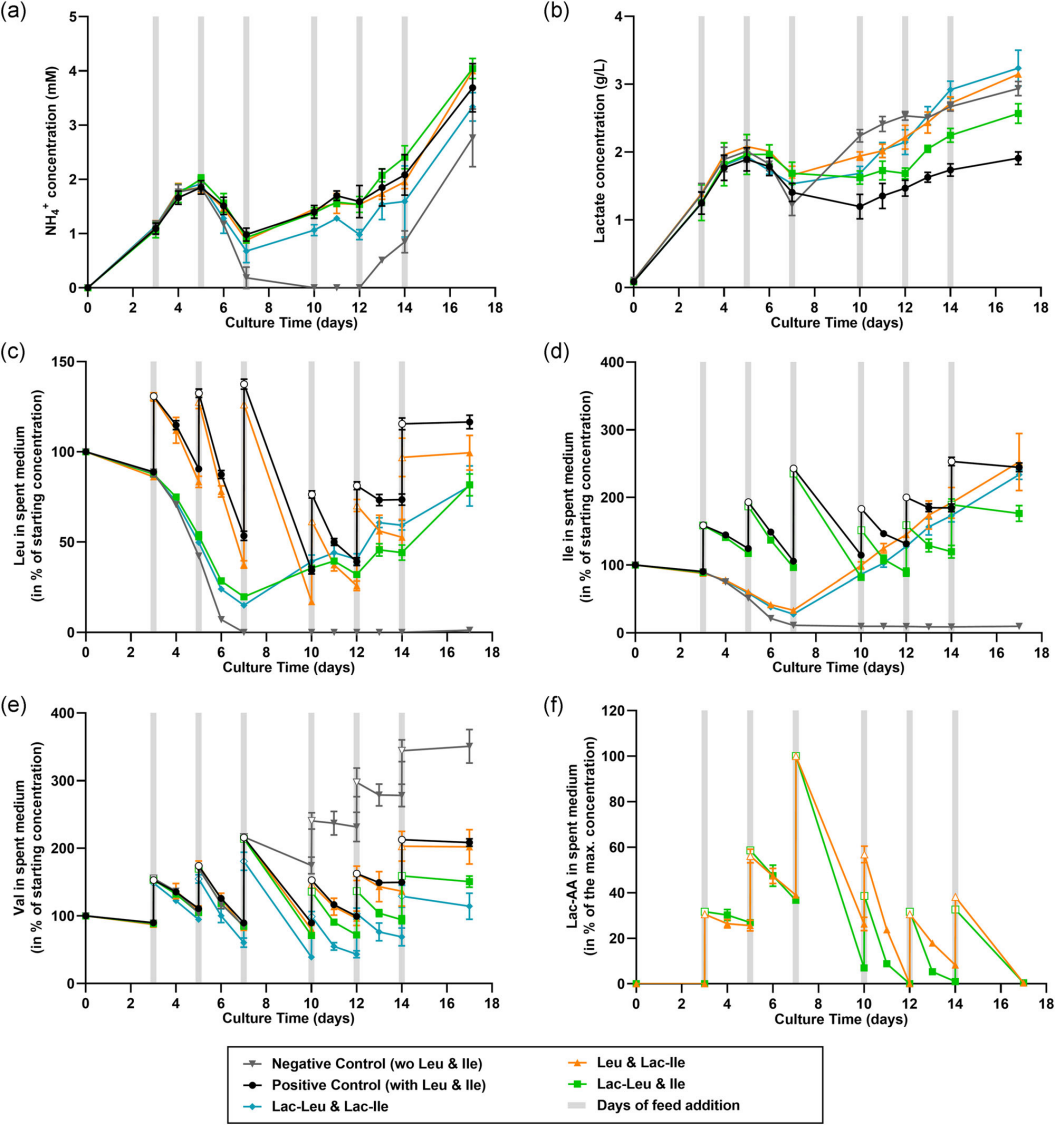
Concentration of metabolites, AA and Lac‐AA in the spent media during the fed‐batch process. (a) NH_4_^+^ and (b) lactate concentrations measured using the Cedex Bio HT (c) Leu, (d) Ile, (e) Val concentrations presented as a percentage of the starting concentration. (f) Lac‐AA concentrations presented as percentage of the maximum concentration. AA concentrations were measured after derivatization using AccQ‐Tag reagent and separation and quantification using UPLC‐UV. Lac‐AA data were obtained using LC–MS. Data are mean ± *SEM* of a minimum of four replicates (metabolites) or two replicates (LC–MS) and were generated from fed‐batch experiments using the CHOK1 GS cell line and feeds containing Lac‐AA. Single points represented with empty symbols in (c)–(f) are calculated values after feeding. AA, amino acids; Ile, isoleucine; Lac‐AA, *N*‐Lactoyl amino acids; LC–MS, liquid chromatography‐mass spectrometry; Leu, leucine; *SEM*, standard error of mean; UPLC‐UV, ultra‐performance liquid chromatography‐ultraviolet; Val, valine [Color figure can be viewed at wileyonlinelibrary.com]

To gain further insights into the mechanism of action of Lac‐AA, important metabolic intermediates were quantified in the spent medium of fed‐batch experiments performed with the CHOK1 GS clone. Ammonia levels were not impacted by the replacement of either Leu or Ile by their respective Lac‐AA. However, a slight overall decrease in the reported NH_3_ values was observed when both AA were replaced simultaneously by Lac‐AA. The negative control showed undetectable levels of ammonia between Day 7 and Day 12, followed by a NH_3_ release from dying/dead cells (Figure 2a). In contrast, cells fed with Lac‐AA released more lactate compared to cells fed with canonical AA, especially after Day 7. The maximum lactate concentration reached 2.5, 3.1, and 3.2 g/L at Day 17 in the Lac‐Leu, Lac‐Ile, and Lac‐Leu and Lac‐Ile conditions, respectively (Figure 2b) whereas only 1.9 g/L lactate was measured in the control condition fed with the canonical amino acids.

Amino acid analysis in spent media showed differences in the concentration of all three BCAA when cells were fed with Lac‐AA (Figure 2c–e). When Leu was replaced with Lac‐Leu, the Leu concentration in the spent medium decreased continuously from Day 0 to Day 5 suggesting that leucine was not or very slowly formed from Lac‐AA during the early exponential phase of the culture (Figure 2c). However, the measured values were higher than the concentrations measured in the negative control after Day 5 (no Leu), indicating that small amounts of Leu can be formed from Lac‐Leu. After Day 7, the Leu concentration in the spent medium increased significantly reaching 81% of the starting concentration at Day 17. The same results were observed upon Lac‐Ile feeding, with a slight release of canonical Ile from Lac‐Ile between Day 5 and 7 followed by a more significant release after Day 7, yielding a final concentration of Ile similar to the control condition at Day 17 (Figure 2d). Interestingly, when replacing Leu with Lac‐Leu, the consumption of Ile and Val was increased, indicating that the metabolism of all three BCAA is interdependent. When Lac‐Leu and Lac‐Ile were used together in the feed, the concentrations of Leu and Ile in the spent medium were similar to the conditions with single replacement, but the overall consumption of Val was increased (Figure 2e). Finally, the kinetics of Lac‐AA consumption was further studied by quantifying Lac‐Leu and Lac‐Ile in the spent medium using targeted LC‐MS. Results presented in Figure 2f confirm the slow consumption of the modified AA during the early exponential phase while the consumption was drastically increased after Day 7, suggesting an active cleavage mechanism, probably mediated by enzymes. This point will be investigated later.

Taken together, data presented in Figures 1 and 2 suggest that Lac‐Leu and Lac‐Ile can substitute canonical Leu and Ile at equimolar ratio in feed with no detrimental effect on cell growth and productivity but with a slight increase in lactate after Day 7. Results also suggest that Lac‐Leu and Lac‐Ile are converted into canonical amino acids mostly during the production phase of the fed‐batch process.

### 3.4 Antibody critical quality attributes in fed‐batch processes using Lac‐AA

**Figure 3:**
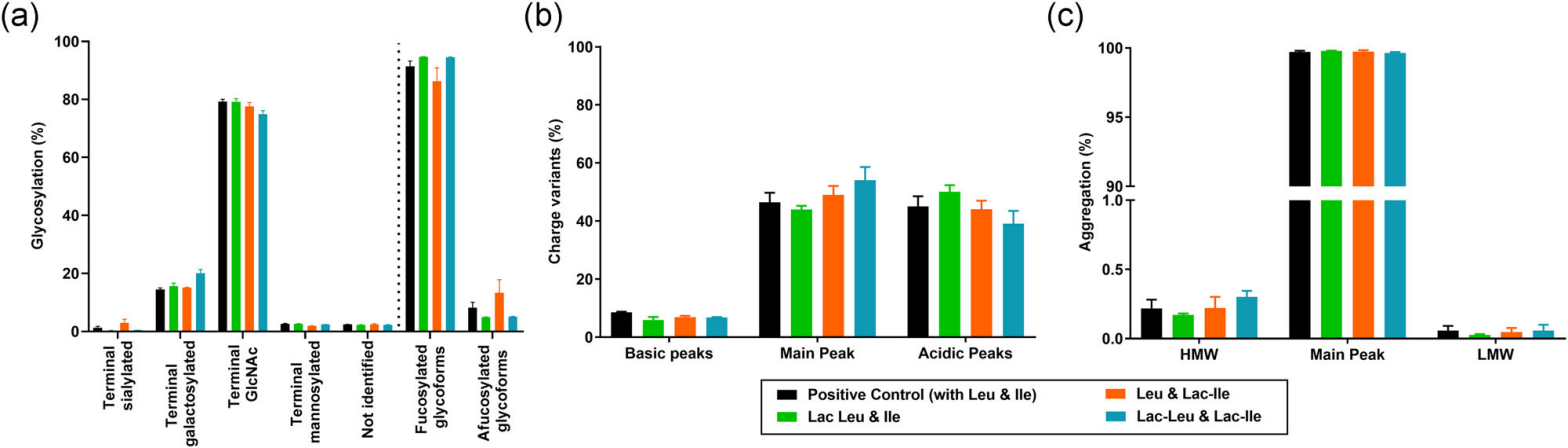
Critical quality attributes of mAb1 produced by the CHOK1 GS cell line fed with canonical and Lac‐AA on Day 12. (a) *N*‐glycosylation measured after APTS labeling and separation and detection using CGE‐LIF on a CESI8000. Results are presented as percentage of terminal sialylated, galactosylated, mannosylated, and *N*‐acetylglucosamine glycoforms. Fucosylated and afucosylated glycans are calculated separately. (b) Charge variants measured using capillary isoelectric focusing on a CESI8000 and presented as percentage of main, acidic, and basic peaks. (c) Percentage of high molecular weight (HMW) and low molecular weight (LMW) forms measured by size exclusion chromatography using a TSK gel Super SW series column and UV detection at 214 nm. Data represent mean ± *SEM* of a minimum of three replicates. CHO, Chinese hamster ovary; Lac‐AA, N‐Lactoyl amino acids; *SEM*, standard error of mean; UV, ultraviolet [Color figure can be viewed at wileyonlinelibrary.com]

In the next step, cQA of mAb1 were studied to understand whether the replacement of Leu and Ile by Lac‐Leu and Lac‐Ile may impact glycosylation, charge variant or aggregation profiles of this IgG1. Results (Figure 3) indicate that all the studied cQA presented comparable levels when produced using either canonical AA or Lac‐AA.

### 3.5 Impact of lactate levels on cell growth and productivity

**Figure 4:**
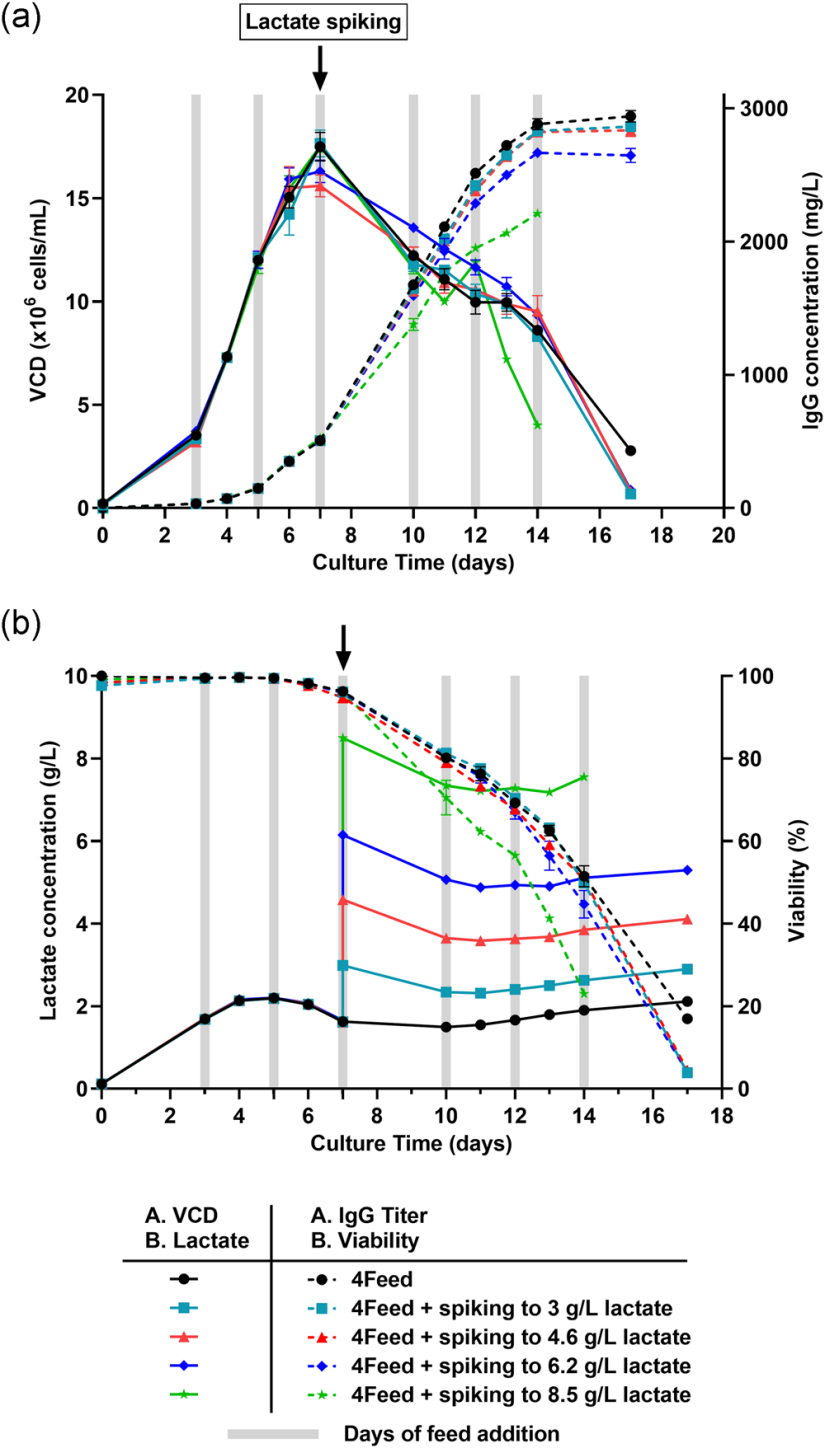
Effect of lactate spiking on cell growth, viability, and titer. (a) Viable cell density (VCD) and titer, (b) viability and lactate obtained in fed‐batch cultivation upon spiking of increasing concentrations of lactate on Day 7. CHOK1 GS were cultivated in Cellvento® 4CHO medium and 3% (v/v) of Cellvento® 4Feed was added on Day 3, 5, 10, 12, and 14 and 6% (v/v) on Day 7. Lactate was measured on Day 7 and increasing amounts of sodium lactate were spiked to the culture to reach 3, 4.6, 6.2, and 8.5 g/L respectively (black arrow). Cultivation was performed in 50 ml spin tubes at 37°C, 5% CO_2_, 80% humidity and a rotation speed of 320 rpm. VCD and viability were measured using Vi‐CELL™ XR and titer and lactate concentrations were measured using Cedex Bio HT. Data represent mean ± *SEM* of four biological replicates. CHO, Chinese hamster ovary; *SEM*, standard error of mean [Color figure can be viewed at wileyonlinelibrary.com]

Since higher lactate concentrations were found in the supernatant during the production phase upon usage of Lac‐AA containing feed (Figure 2b), the impact of lactate spiking on the overall performance was investigated. Indeed, high concentrations of lactate in spent media during fed‐batch bioprocesses are known to have detrimental effects on cell growth and IgG titer (Freund & Croughan, 2018; Hartley et al., 2018; Zagari et al., 2013). With the CHOK1 GS clone, increasing amounts of exogenous lactate were spiked into the cell culture at Day 7, at the beginning of the production phase. Results (Figure 4) indicate that concentrations up to 4.6 g/L lactate on Day 7 had no effect on either VCD, viability or IgG titer, while 6.2 g/L lactate (or higher) had an effect of overall productivity. Indeed, the IgG titer on Day 14 was reduced significantly by 22% in the condition where 8.5 g/L lactate was spiked on Day 7. Altogether, these data suggest that the up to 3.2 g/L lactate found upon Lac‐AA feeding are unlikely to affect process performance.

### 3.6 Optimization of Lac‐AA concentrations to improve IgG yield

**Figure 5:**
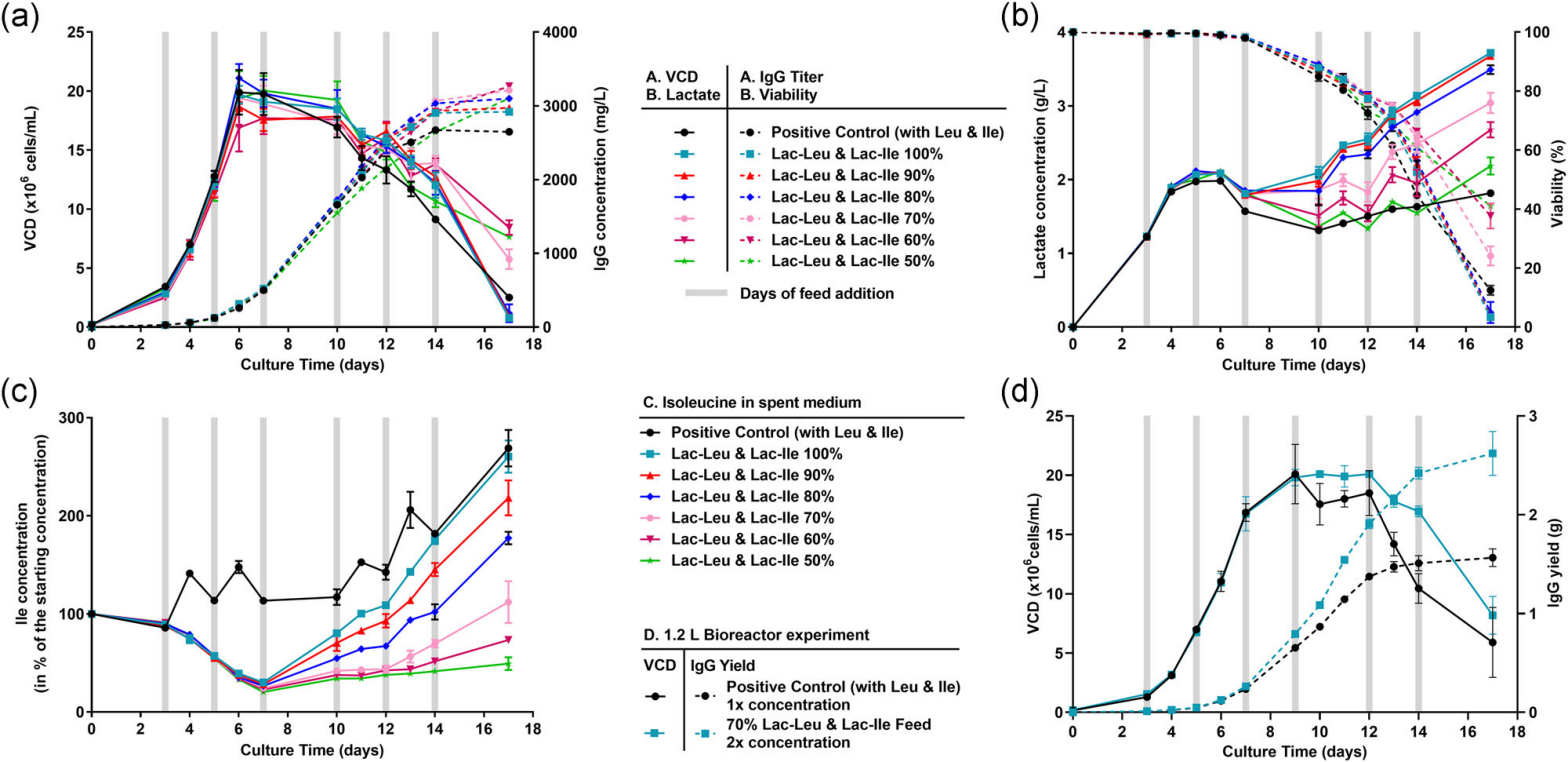
Optimization of Lac‐AA concentrations and concentrated bioreactor process. (a) Viable cell density and titer, (b) viability and lactate concentration, and (c) relative Ile concentration obtained in fed‐batch with CHOK1 GS clone fed with 100% canonical Leu and Ile or decreasing concentrations of Lac‐Leu and Lac‐Ile (up to 50%). (d) VCD and IgG yield obtained in a bioreactor fed‐batch experiment using the control feed at 1× concentration and a 2× concentrated feed containing 70% of Lac‐Leu and Lac‐Ile. In the experiment shown in (a)–(c), CHOK1 GS were cultivated in Cellvento® 4CHO medium and 3% (v/v) of Cellvento® 4Feed was added on Day 3, 5, 10, 12, and 14 and 6% (v/v) on Day 7. Cultivation was performed in 50 ml spin tubes at 37°C, 5% CO_2_, 80% humidity and a rotation speed of 320 rpm. For the experiment shown in (d), the volume of Lac‐AA containing feed added was reduced by 2× due to the higher overall concentration. The fed batch was performed in 1.2 L bioreactors controlled at 37°C, pH 6.95 ± 0.15, 50% dissolved oxygen and agitation at 140 rpm. VCD and viability were measured using Vi‐CELL™XR, and titer and lactate were measured using Cedex Bio HT. Ile concentrations were measured after derivatization using AccQ‐Tag reagent and separation and quantification using UPLC‐UV. Data represent mean ± *SEM* of four replicates in spin tubes and two bioreactors. CHO, Chinese hamster ovary; Ile, isoleucine; Lac‐AA, *N*‐Lactoyl amino acids; Leu, leucine; *SEM*, standard error of mean; UPLC‐UV, ultra‐performance liquid chromatography‐ultraviolet [Color figure can be viewed at wileyonlinelibrary.com]

The next experiment sought to determine the optimal concentration of Lac‐AA in the feed. The aim was to reduce the quantity of required raw materials, circumvent the slightly higher lactate production and generate a highly concentrated feed. Results presented in Figure 5 indicate no change in VCD or titer for Lac‐AA concentrations between 60% and 100% of the concentrations of the canonical AA. In contrast, reducing the Lac‐AA concentration by 50% resulted in a slightly lower titer after Day 7. The viability in all the Lac‐AA conditions was higher than the viability in the control condition (Figure 5b). When considering the released lactate (Figure 5b) and Ile concentrations (Figure 5c), the decrease in Lac‐AA in the feed led to a dose dependent decrease of the concentration of both molecules after Day 7. A concentration of 70% Lac‐AA was chosen for the subsequent bioreactor experiments.

To demonstrate that the high solubility of Lac‐AA enables the development of highly concentrated feed formulations that can be added to the process through lower volumes, a bioreactor experiment was designed. The control condition was identical to the control condition in spin tube. The Lac‐AA containing feed contained only 70% of the Lac‐AA (compared to the canonical AA) and was concentrated two times compared with the control condition. Consequently, two times less volume was added on the respective feeding days, thus reducing the diluting effect of the feed on the product. To maximize the bioreactor utilization using a concentrated feed, the Lac‐AA process started with a 12% higher volume compared to the control condition. Since the same seeding density was used, this lead to a higher absolute amount of cells inoculated at Day 0 in the concentrated process. Results (Figure 5d) indicate that the VCD was maintained at higher levels after Day 9 in the Lac‐AA condition. Interestingly, the addition of a concentrated feed in a process starting with higher medium volume and thus higher absolute amounts of cells afforded an increase in the IgG yield by 58% on Day 14, suggesting the productivity benefits can be obtained using concentrated processes.

### 3.7 Mechanism of Lac‐AA cleavage

**Figure 6:**
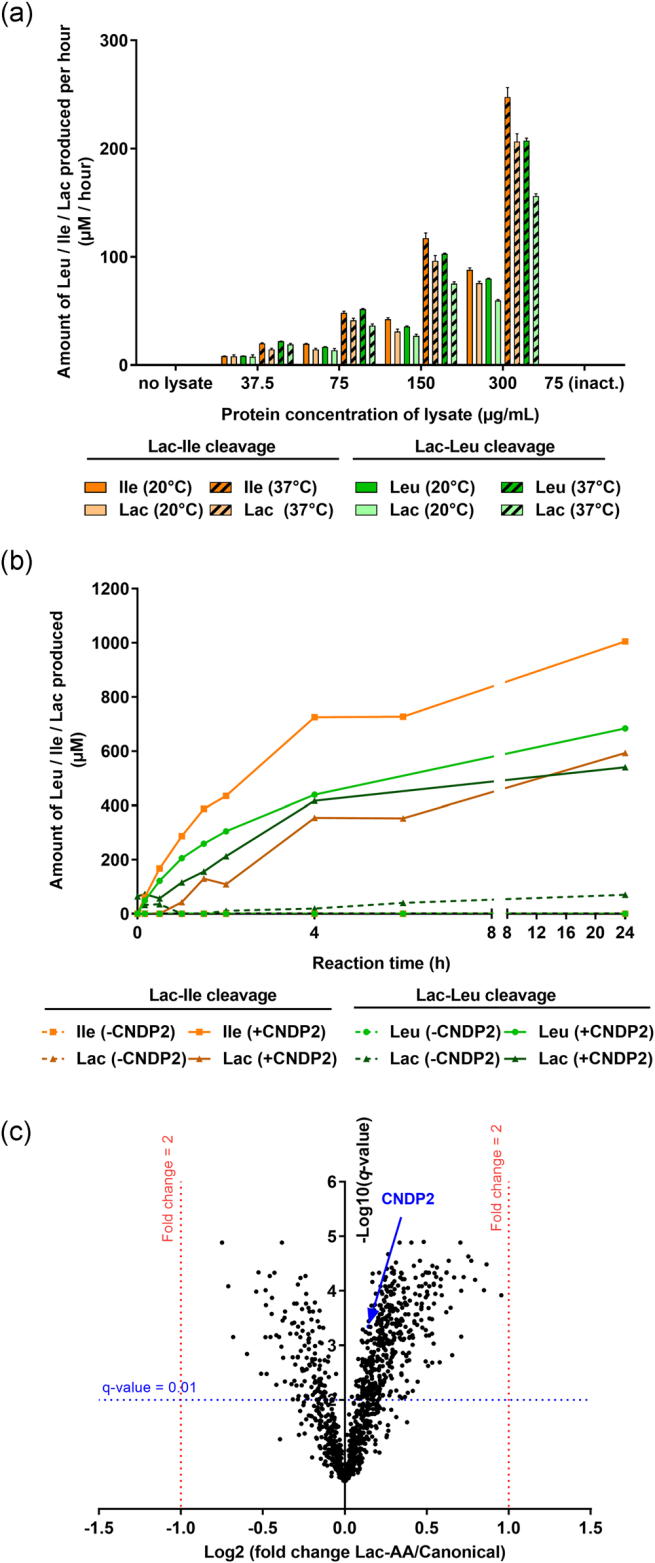
Mechanism of Lac‐AA cleavage. (a) CHO lysate mediated Lac‐AA cleavage: Protein concentrations were quantified in CHOK1 GS lysates and increasing protein amounts were incubated with either 25 mM Lac‐Leu or 25 mM Lac‐Ile at 20°C or 37°C. A pre‐incubation of the lysate at 100°C for 10 min was also performed to inactivate enzymes. Values represent the amount of Leu, Ile, or lactate produced per hour. (b) CNDP2 mediated *N*‐Lactoyl‐amino acid cleavage. 1 µg CNDP2 enzyme was incubated with 10 mM of the Lac‐Ile and Lac‐Leu at 37°C and samples were collected at the indicated time points. Controls were incubated in the same conditions in the absence of enzyme. Lactate levels were measured using the lactate fluorescent kit, whereas Leu and Ile concentrations were measured after derivatization using AccQ‐Tag reagent and separation and quantification using UPLC‐UV. (c) Label free LC‐MS based proteomic study comparing CHOK1 GS lysates from a Lac‐AA and a control process. The *x*‐axis shows the log2 of protein fold‐change in the Lac‐AA condition compared to control, whereas the *y*‐axis shows the −log10 of the calculated *q* value (adjusted *p* value from Progenesis QI). Only proteins quantified with at least three unique peptides were considered (1179 proteins). Proteins matching the following criteria: *x* > |1| and *y* > 2 were considered as statistically significant (*q* value < 0.01). CNDP2 is highlighted in blue in the plot. CHO, Chinese hamster ovary; Ile, isoleucine; Lac‐AA, N‐Lactoyl amino acids; Leu, leucine; UPLC‐UV, ultra‐performance liquid chromatography‐ultraviolet [Color figure can be viewed at wileyonlinelibrary.com]

To investigate whether the release of canonical AA from Lac‐AA is mediated by enzymes, both modified AA were incubated with cell lysates from CHOK1 GS clone and the release of Leu, Ile, and lactate was monitored over time. Results indicate that the release of both lactate and canonical AA was linear with time (not shown) and was dependent on the protein concentration of the lysate (Figure 6a). The release was higher at 37°C compared with RT and was completely inhibited when lysates were pre‐incubated at 100°C, supporting the hypothesis of an enzyme mediated Lac‐AA cleavage. The release of lactate and the respective canonical AA was recorded with a stochiometric ratio close to 1:1, even though the lactate concentrations were always slightly lower than the AA concentrations probably indicating a further oxidation to, for example, pyruvate.

As was published elsewhere (Jansen et al., 2015), the protein CNDP2 enables the synthesis of *N*‐lactoyl‐phenylalanine when incubated with its precursors phenylalanine and lactate. Since this enzyme is reported as a nonspecific dipeptidase, we hypothesized that it may also cleave Lac‐Leu and Lac‐Ile. In fact, release of lactate and respective free AA (i.e., Leu and Ile, Figure 6b) was observed upon incubation of lac‐AA with recombinant CNDP2 enzyme. The reaction was time dependent but nonlinear and reached saturation after 6 h of incubation.

Finally, to confirm that CNDP2 is expressed by CHO cells, a proteomic characterization of a CHO GS cell lysate was performed. A sample collected on Day 10 from the control process was compared to a sample collected on the same day from the Lac‐AA process. About 1179 proteins were quantified with at least three unique peptides (Table S2). CNDP2 was identified in both samples at the same level confirming that CHO cells express this dipeptidase, and suggesting that the amount of endogenous CNDP2 might be sufficient to cleave Lac‐AA. When looking at the entire data set using a volcano plot (Figure 6c), no protein was over‐ or underexpressed in the Lac‐AA condition compared with the control (fold threshold of 2, *q* value < 0.01), suggesting no major change at the protein level upon usage of Lac‐AA. Altogether, these results suggest that Lac‐AA are very likely cleaved by intracellular enzymes such as CNDP2, a dipeptidase proved to be expressed by CHO cells. CNDP2 was able to cleave Lac‐AA in vitro as shown in Figure 6b and is thus likely to at least participate in the release of bioavailable canonical AA in cell culture.

## 4 DISCUSSION

This study sought to investigate the possibility to replace Leu and Ile in CCM formulations by their respective Lac‐AA counterparts, Lac‐Leu and Lac‐Ile. Lac‐AA are relatively new molecules, discovered by the food industry at the end of the 1990s, and especially known for their taste properties in cheese (Frerot & Escher, 1998). Results presented in this study indicate that the replacement of Leu and Ile by the derivatives enables the development of highly concentrated feed formulations due to the higher solubility of Lac‐AA when compared to canonical AA. Both modified AA were shown to be stable in a complex CCM formulation when stored at 4°C and RT for at least 3 months. While very little is reported in the literature about the chemical stability of Lac‐AA, several studies report that Lac‐AA are very resistant to degradation even in the presence of exoproteases and endoproteolytic enzymes such as trypsin, chymotrypsin, pepsin, and carboxypeptidase Y (Bottesini et al., 2014; Lorbert et al., 2004; Sforza et al., 2009).

When used in fed‐batch processes, the 1:1 molar exchange of either Leu or Ile with Lac‐Leu or Lac‐Ile led to a comparable productivity and cell growth as in the control process, indicating that Lac‐AA are bioavailable for CHO cells. Furthermore, the use of Lac‐AA led to a dose dependent increase of lactate in the spent medium, as a result of the cleavage of the molecule. In the literature, numerous studies reported an inhibition of cell growth and productivity due to an excess of lactate in the CCM (Hassell et al., 1991; Lao & Toth, 1997), however fairly recent studies have demonstrated that a significant fraction of the negative effects of lactate may be caused by the increase in osmolality resulting from the neutralization of lactic acid during pH control (Buchsteiner et al., 2018). Our spiking study confirmed that concentrations of lactate up to 4.6 g/L have no significant negative effect on overall productivity when added at Day 7 of the culture. Furthermore, it is worth to point out that the release of lactate from Lac‐AA starts mainly after Day 7 which is often the timepoint when cells start shifting their metabolism towards lactate consumption. Thus, lactate released specifically during the production phase might be beneficial to cells and may serve as a source of three carbon intermediates enabling the uncoupling of the mitochondrial energy generation from the glycolysis as suggested by Rabinowitz and Enerback (2020) and demonstrated for CHO cells by Li et al. (2012). In case the lactate concentration proves to be too high for some specific clones, different strategies can be used to promote the metabolic shift, including: replacing glucose with alternative sugars (Altamirano et al., 2004), maintaining glucose or glutamine concentrations to low levels using online feedback control (Gagnon et al., 2011), performing a pH shift or a temperature shift to lower values (Ivarsson et al., 2015; Zalai et al., 2015), adapting cell lines to higher lactate concentrations (Freund & Croughan, 2018) or supplementing the media or feeds with copper (Luo et al., 2012; Qian et al., 2011; Yuk et al., 2015). Altogether, the concentration of lactate released upon feeding with Lac‐AA is low and many strategies are available to alleviate a potential increase of this metabolite.

Mechanistic studies aiming at understanding how CHO cells utilize Lac‐AA indicated that a very small amount of canonical AA was released in the supernatant during the exponential phase of the culture, while increasing amounts were released during the production phase. This kinetic was very similar to the kinetic of tyrosine release from phosphotyrosine, which is known to be mediated by cytosolic phosphatases (Zimmer et al., 2014). Based on previous studies showing that CNDP2 is capable of producing Lac‐AA (Jansen et al., 2015), the results presented in this study highlight that Lac‐AA are cleaved by enzymes released by CHO cells and at least in part, by CNDP2. This cytosolic nonspecific dipeptidase is a metallopeptidase of the M20 family (uses Mn or Zn as a cofactor) and is known in humans to hydrolyze dipeptides including l‐carnosine or Cys‐Gly (Kaur et al., 2009; Teufel et al., 2003). While its main function is likely the hydrolysis of dipeptides containing hydrophobic AA, the reverse proteolysis was demonstrated to be dependent on the substrate concentrations in specific tissues (Jansen et al., 2015). Obviously, several other enzymes may also be involved in the cleavage of the Lac‐AA N‐terminal peptide bond such as common cytosolic aminopeptidases (e.g., Uniprot A0A3L7HBS8, which was detected in CHO cells, Table S2) or aminopeptidases with a different predicted subcellular localization (e.g., Uniprot A0A3L7I5X6, G3H3H9, also detected in this proteomic study, Table S2). More specific BCAA aminopeptidases such as Lap3 may also be good candidates for the cleavage since they are described for their active hydrolysis of Leu containing peptides. The homologous protein of Lap3 in CHO is described as Cysteinylglycine‐S‐conjugate dipeptidase (uniprot A0A098KXB1, 92.1% homology with human LAP3). However, the detailed investigation of these peptidases is beyond the scope of the current manuscript.

Finally, the higher solubility of Lac‐AA enabled the development of a concentrated feed formulation, which was applied successfully in bioreactors allowing an increase in process efficiency and a better bioreactor utilization. The use of concentrated CCM is important for applications such as inline dilution of media allowing to reduce the manufacturing footprint. In fed‐batch, the higher IgG yield obtained in this study was likely due, at least in part, to the reduction of the volume of liquid added throughout the process and the possibility to start the culture with a higher volume of medium. This demonstrated the possibility to improve the overall performance by optimization of the bioreactor space time yield using a concentrated process.

Concluding, this report shows for the first time the successful application of Lac‐Leu and Lac‐Ile to increase the overall concentration of CCM formulations used to produce antibodies or recombinant proteins based on CHO expression systems. The use of these Lac‐AA enables the development of next generation CCM formulations and provides new opportunities to reduce the feeding volume and increase the recombinant protein yield. Like for any novel raw material used in bioprocesses, this study furthermore highlights the importance of a profound knowledge of the molecular mechanism of action to design optimal CCM formulations. In the future, approaches for alternative highly soluble and bioavailable AA derivatives, as successfully shown in this study, could support rapid pharmaceutical production of complex biotherapeutics.

## AUTHOR CONTRIBUTIONS

Corinna Schmidt and Dmitry Zabezhinsky performed cell culture experiments, stability and solubility experiments as well as the corresponding analytic. Dmitry Zabezhinsky wrote the initial manuscript. Alisa Schnellbaecher performed the small‐scale bioreactor experiments. Maria Wehsling performed the analysis of cell culture media components and cQAs of the recombinant proteins. Maxime Le Mignon designed and performed all LC‐MS related studies (small molecules and proteins). Gregor Wille, Yannik Rey and Markus Fischer developed the synthesis process for lactoyl‐amino acids and produced them at scale; Aline Zimmer designed, supervised the study, and reviewed the data and the manuscript. All authors have approved the final article.

## Supporting information

Supporting information.

## Data Availability

Data available in article supplementary material.
