# Supplementary material for: Lactoyl leucine and isoleucine are bioavailable alternatives for canonical amino acids in cell culture media
Source: Biotechnol Bioeng. 2021 Apr 8;118(9):3395–408. doi: 10.1002/bit.27755 (PMC8453549; doi:10.1002/bit.27755)
Supplement: Supplementary file 1 — Supporting information. [file BIT-118-3395-s001.docx]

**Supplementary information**

Lactoyl leucine and isoleucine are bioavailable alternatives for canonical amino acids in cell culture media

Corinna Schmidt^a^, Maria Wehsling^a^, Maxime Le Mignon^a^, Gregor Wille^b^, Yannick Rey^b^, Alisa Schnellbaecher^a^, Dmitry Zabezhinsky^a^, Markus Fischer^b^, Aline Zimmer^a*^

**Supplementary Table 1:** Solubility (g/kg) of Leu and Ile and their derivatives in water at different temperatures. The solubility was determined using saturated solutions and residual mass determination, the pH was recorded and is displayed in bracket.

| Solubility at given temperatures, in g/kg (pH) | | | | |
| --- | --- | --- | --- | --- |
|  | 4 °C | 20 °C | 25 °C | 37 °C |
| Leucine | 20.6 (6.1) | 21.5 (5.7) | 22.1 (6.0) | 23.2 (5.8) |
| Leucine sodium salt | 81.4 (11.2) | 81.6 (10.8) | 86.0 (10.8) | 149.8 (11.0) |
| Lactoyl Leu sodium salt | 633.3 | 640.0 | 689.2 (6.7) | 744.5 |
| Isoleucine | 29.5 (6.4) | 31.5 (6.4) | 32.4 (6.2) | 34.0 (6.1) |
| Lactoyl Ile sodium salt | 577.7 | 604.8 | 639.3 (6.1) | 685.5 |

**Supplementary Table 2: Proteins identified using label free LC-MS in CHOK1 GS cell lysates obtained at D10 of a fed-batch process using either the control or the Lac-AA containing feed.** Proteins were identified with at least 3 unique peptides, CNDP2 is marked in bold.

**Supplementary Figure 1:** Chemical structure of Lac-Leu and Lac-Ile sodium salts and NMR and MS characterization data.

**N-Lactoyl-leucine** sodium salt**.** ^1^H NMR: δ 0.88 (d, *J* = 6.1 Hz, 3H, CH(C*H*_3_)_2_), 0.91 (d, *J* = 6.1 Hz, 3H, CH(C*H*_3_)_2_), 1.36 (d, *J* = 6.9 Hz, 3H, OCHC*H*_3_), 1.54-1.67 (m, 3H, NCHC*H_2_*CH and NCHCH_2_C*H*), 4.19-4.25 (m, 1H, NC*H*), 4.25 (q, *J* = 6.8 Hz, 1H, OC*H*) ppm; ^13^C NMR: δ 19.9, 21.2, 22.8, 24.9, 41.1, 53.7, 68.1, 177.1, 180.1 ppm; ESI-MS: [M+H]^+^ = 204.1234 (C9H18NO4), main MSMS fragments: 86.096, 132.102, 158.118; [M-H]^-^ = 202.1085 (C9H16NO4), main MSMS fragments: 114.092, 130.087, 140.108, 158.119

**N-Lactoyl-isoleucine sodium salt.** ^1^H NMR: δ 0.82 (t, *J* = 5.6 Hz, 3H, CH_2_C*H*_3_), 0.85 (d, *J* = 6.8 Hz, 3H, CH_2_CHC*H*_3_), 1.13 (sept, *J* = 7.2 -7.6 Hz, 3H, C*H*_2_CH_3_), 1.32 (d, *J* = 6.9 Hz, 3H,C(OH)HC*H*_3_), 1.37 (m, 3H, C*H*_2_CH_3_), 1.82 (m, 3H, CH_2_C*H*CH_3_), 4.06 (d, 1 H, *J* = 5.8 Hz, C*H*CN), 4.21 (q, 1H, *J* = 6.9 Hz, C(OH)*H*CH_3_) ppm; ^13^C NMR: δ 13.3, 17.8, 22.1, 26.9, 39.6, 61.8, 70.2, 179.2, 180. 8 ppm; ESI-MS: [M+H]^+^ = 204.1234 (C9H18NO4), main MSMS fragments: 86.096, 132.102, 158.118; [M-H]^-^ = 202.1085 (C9H16NO4), main MSMS fragments: 114.092, 130.087, 140.108, 158.119





**Supplementary Figure 2: Determination of the maximum solubility of Lac-Leu and Lac-Ile in Cellvento® 4Feed.** A. Solubility of Leu and Lac-Leu and B. Ile and Lac-Ile in Cellvento® 4Feed lacking Ile and Leu (125 g/L, pH 7.0 ± 0.2, measured at room temperature). Increasing amounts of the Lac-AA (or AA) were added successively to the feed until saturation was observed. After each addition, the feed was agitated for 10 min, and turbidity was measured using Turb® 550 IR (WTW Lab). A solution having a turbidity below 5 NTU was considered soluble. The limit of 5 NTU is represented in the graph by a horizontal dotted line.

**Supplementary Figure 3: Stability of Lac-Leu and Lac-Ile in Cellvento® 4Feed.** A. Stability of Lac-Leu and B. Lac-Ile in Cellvento® 4Feed lacking Ile and Leu and stored for up to 90 days at either 4 °C or RT light protected. Quantification was performed using LC-MS with detection of the [M-H]^-^ of both Lac-AA in ESI negative mode. Data represent mean ± SD of 2 replicates.
